# Liver disease referrals to an urban, hospital‐based hepatology outpatient clinic over the past 25 years

**DOI:** 10.1002/jgh3.12286

**Published:** 2019-12-05

**Authors:** Micah Grubert Van Iderstine, Daniel Iluz‐Freundlich, Casandra Dolovich, Eurielle Villarin, Gerald Y Minuk

**Affiliations:** ^1^ Section of Hepatology, Department of Internal Medicine University of Manitoba Winnipeg Manitoba Canada; ^2^ Department of Pharmacology and Therapeutics, Rady College of Medicine University of Manitoba Winnipeg Manitoba Canada

**Keywords:** alcoholic liver disease, hepatitis B, hepatitis C, hepatology, liver disease, non‐alcoholic fatty liver disease, primary biliary cholangitis, primary sclerosing cholangitis

## Abstract

**Background:**

Additional hepatologists are required to manage the rapidly increasing number of patients with liver disease. One disincentive to trainees considering a career in hepatology is the longstanding perception that outpatient hepatology consists largely of managing patients with alcohol‐induced liver disease (ALD).

**Objectives:**

To document the types of liver diseases and changes in liver disease referrals to an urban outpatient liver disease clinic over the past 25 years.

**Methods:**

The nature of the liver disorder, age, gender, and socioeconomic status of patients referred to an urban, hospital‐based, liver diseases outpatient program were documented from 1992 to 2017. Joinpoint analysis was performed to identify significant trends in referral prevalence rates of various disorders.

**Results:**

In 1992/1993, hepatitis C virus (HCV), followed by hepatitis B virus (HBV), “other”, non‐alcoholic fatty liver disease (NAFLD), and primary biliary cholangitis (PBC) were the most common underlying liver diseases in referred patients (39, 36, 12, 4.5, and 3.5% respectively), whereas in 2016/2017, NAFLD, HBV, HCV, “other,” and ALD were most common (60, 15, 12, 8.7, and 3.3%, respectively). Aside from NAFLD referrals, which consistently increased over the 25‐year period, the prevalence of all other liver disease referrals fluctuated but generally declined. Recently referred patients were significantly older (38 ± 13 years in 1992/1993 and 49 ± 15 years in 2016/2017, *P* < 0.0001), while gender and socioeconomic status have not changed.

**Conclusions:**

Hepatology is a diverse, dynamic subspecialty where ALD continues to constitute less than 5% of all patient referrals.

## Graphical abstract

Outpatient hepatology consists of a variety of liver disorders. Over the past 25 years, the prevalence of these disorders has significantly changed. Such information is very useful for trainees considering a career in hepatology.

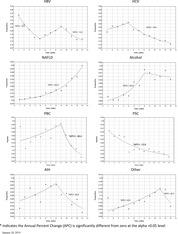

## Introduction

Hepatology is a subspecialty of Internal Medicine that has developed rapidly since the early 1980s when liver transplantation was designated a nonexperimental treatment option for patients with advanced liver disease and more recently, in response to increases in the prevalence of viral hepatitis and non‐alcoholic fatty liver disease (NAFLD).^1^, ^2^, ^3^, ^4^ Accompanying the evolution of hepatology has been the need for additional physicians with expertise in the discipline. One of the challenges in attracting trainees to a career in hepatology has been the misperception that alcohol‐induced liver disease (ALD) constitutes the vast majority of liver disease they will encounter in their clinical practice. In response to that challenge, in 1996, we described the prevalence of liver diseases seen in 1226 adult patients referred to our urban, hospital‐based outpatient liver clinic.^5^ In descending order, hepatitis B virus (HBV), hepatitis C virus (HCV), NAFLD, drug‐induced liver injury (DILI), ALD, primary biliary cholangitis (PBC), primary sclerosing cholangitis (PSC), and autoimmune hepatitis (AIH) were the most common indications for referral, followed by 34 other acute or chronic liver disorders.

Over the past 25 years, risk factors and treatments for many types of acute and chronic liver disease have changed. These changes are likely to have impacted the profile of liver diseases seen in outpatient clinics. Thus, in the present study, we documented the prevalence rates of the most common causes of liver disease within our referred patient population and changes to the prevalence rates over the past 25 years. We also determined whether any changes observed were gender specific and/or associated with changes in the age or socioeconomic status of referred patients.

## Methods

### *Database and patient population*

The database used for this retrospective analysis the Philip and Ellie Kives Clinical Database. Over the past 30 years, patients referred to the clinic have had their demographic data, laboratory findings, and radiologic and liver biopsy reports entered into the database. Specific diagnoses have also been entered but more sporadically and only when standard diagnostic criteria for each condition are met.

For the purpose of this study, database and administrative billing files were searched for all patients with an entered diagnosis of hepatitis B (acute or chronic), hepatitis C (acute or chronic), NAFLD (fatty liver or non‐alcoholic steatohepatitis), ALD (alcohol‐induced hepatitis or cirrhosis), PBC, PSC, or AIH and the year of their initial visit from 1992 to 2017 inclusive. The year 1992 was considered the initial year for the study to allow for referral biases that might occur during the initial 5 years of a newly offered liver diseases program. In patients with more than one cause of liver disease, each condition was considered separately. There were no exclusion criteria. The data retrieved from each file included underlying liver diagnosis, age, gender, postal code, and year of initial referral visit.

Socioeconomic status was determined by the patient's postal code and postal code‐matched median family income (2006 census) data from Statistics Canada. Together, this input allowed patients to be considered living in low‐, medium‐, or high‐income areas.

### *Statistical analysis*

All statistical analyses were conducted using SAS 9.3 for Windows. Univariate analysis was conducted using Student's *t*‐test for continuous variables and Chi‐square test for categorical variables with more than two levels. Joinpoint regression analysis was performed in order to identify statistically significant changes in trends in the proportion of liver disease referrals out of total referred patient population, where joinpoint regression, in this case, tests the hypothesis that the trend in the proportion of liver disease is neither increasing nor decreasing (i.e. H_0_: The APC = 0 *vs* H_A_: The APC ≠ 0), where APC is the annual percent change and can be rejected using alpha at the 5% level.

## Results

**Figure 1:**
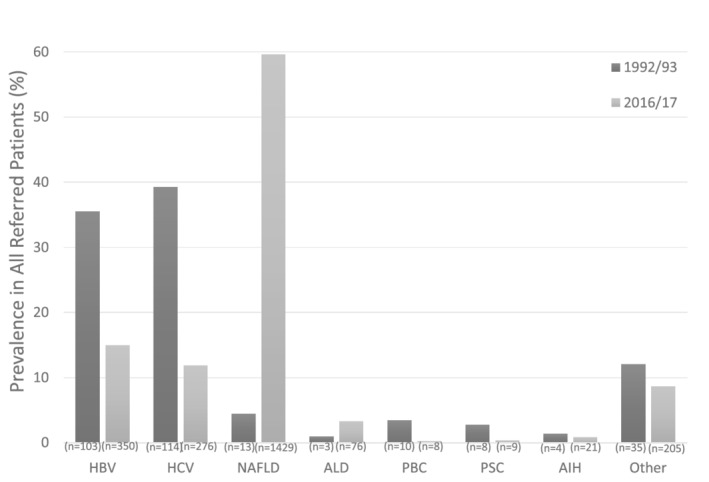
Changes in the prevalence rates of various liver disorders as a percentage of total referred patients to an urban liver diseases outpatient clinic over a 25‐year period. See text for description of abbreviations.

A total of 13 465 patients had a specific diagnosis entered between 1992 and 2017. Figure 1 outlines the prevalence of each condition relative to the total number of referred cases and the absolute number of referrals at the beginning and end of the study period. In 1992/1993, HCV (40%), HBV (36%),“other” (12%), NAFLD (4.5%), PBC (3.5%), PSC (2.8%), AIH (1.4%), and ALD (1.0%) were the most common causes of liver disease in referred patients, whereas in 2016/2017, NAFLD (60%), HBV (15%), HCV (12%), “other” (8.7%), ALD (3.3%), AIH (0.9%), PSC (0.4%), and PBC (0.3%) were the most common. Patients with “other” diagnosis had one or more of 39 liver conditions similar to those outlined in the 1996 report.^5^

**Figure 2:**
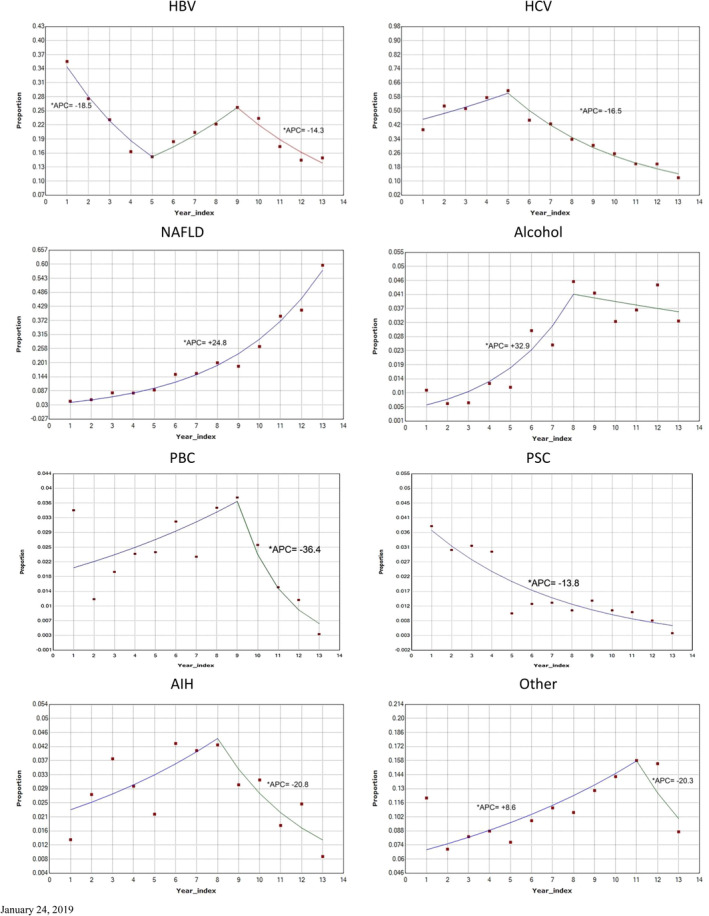
Joinpoint analyses of trends in liver disease referrals as percentage of total referred patient population. Only statistically significant annual percent changes (APCs) are indicated. Year index refers to data derived from consecutive 2‐year periods over the 25‐year study period. See text for description of abbreviations. *Indicates the Annual Percent Change (APC) is significantly different from zero at the alpha = 0.05 level

Figure 2 provides the prevalence rates of each condition amongst the total referred patient population from 1992 to 2017 expressed in 2‐year intervals. The only consistent increase in prevalence that occurred over the entire time period was in NAFLD patients with an APC of 24.8% per year. The remaining conditions consisted of fluctuating prevalence rates with significant increases and decreases. HBV was the only condition that showed multiple significant trends during two time periods, decreasing from 1992/1993 to 2000/2002 (APC = −18.5%, *P* < 0.05) and 2008/2009–2016/2017 (APC = −14.3%, *P* < 0.05). In addition to NAFLD, significant increases were observed in patients with ALD between 1992/1993 and 2006/2007 (APC 32.9%) and “other” liver diseases between 1992/1993 and 2012/2013 (APC 8.6%). Significant decreases were observed in patients with PBC between 2008/2009 and 2016/2017 (APC = −34.4%), AIH between 2006/2007 and 2016/2017 (APC = −20.8%), HCV between 2000/2001 and 2016/2017 (APC = −16.5%), and PSC between 1992/1993 and 2016/2017 (APC = −13.0%).

**Figure 3:**
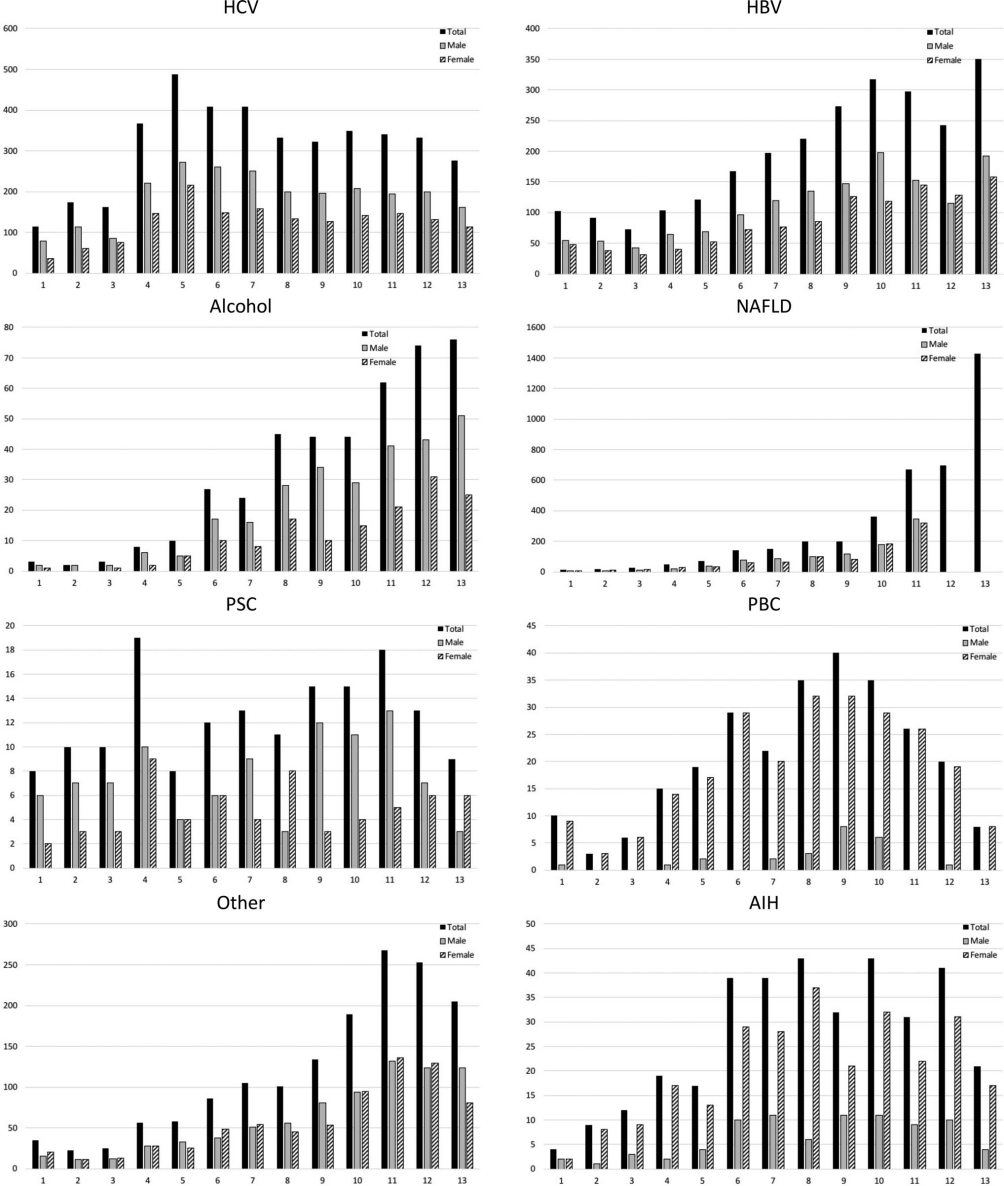
Absolute number of patients (total, male, and female) with specific liver disorders referred to an urban liver diseases outpatient clinic over a 25‐year period. See text for description of abbreviations.

Figure 3 provides the absolute number of patient referrals in each 2‐year interval and gender distributions for each condition over the 25‐year time period. In general, the actual numbers of NAFLD, HBV, ALD, and “other” patient referrals increased throughout this time period, whereas HCV, PBC, and AIH increased until 2000–2010 and subsequently declined. The number of PSC referrals had not changed over the 25 year period.

Male referrals were more common for HBV, HCV, ALD, and PSC, whereas females predominated in PBC and AIH referrals. Male and female referrals were similar for NAFLD and “other” liver disorders. Aside from an increase in HCV‐infected females (1992/1993: 31% *vs* 2016/2017: 41%, *P* = 0.05), there were no significant changes in gender distribution over time for the remaining liver disorders.

Table 1 describes the mean (±SEM) ages and socioeconomic status of referred patients for each condition over the study period. In general, patients referred toward the end of the study period were approximately 10 years older than patients referred initially (*P* < 0.0001). This pattern applied to most disease types and was gradual over the 25‐year period. Although HCV patients resided in lower‐income areas, the socioeconomic status of these and other patients had not significantly changed over the past 25 years, with approximately 40% of patients residing in low‐, 50% middle‐, and 10% high income areas throughout the study period.

**Table 1:** Mean ages and socioeconomic status of study patients in 1992/1993 and 2016/2017

	1992/1993	2016/2017
Age in years (±SEM)	38 (14)	49 (15)
Socioeconomic status (%)		
Low	64 (40)	432 (38)
Middle	75 (47)	588 (51)
High	21 (13)	125 (11)

## Discussion

This study describes the prevalence of common liver diseases referred to an urban, hospital‐based, liver diseases outpatient clinic over a recent 25‐year period. The results indicate that, although HCV was the most common indication for patient referral in 1992/1993 (39%), in 2016/2017, NAFLD referrals were most common (60%). Moreover, ALD continues to be a relatively infrequent cause for patient referral (1% in 1992/1993 and 3.3% in 2016/2017). The results also reveal that trends in referrals are not being driven by changes in gender or socioeconomic status of referred patients, but recently referred patients are approximately 10 years older than those referred in the early 1990s.

The exponential increase in NAFLD rates parallels increases in the prevalence of obesity and diabetes (the two most common risk factors for NAFLD) within the general population over the same time period.^6^ In addition, the acquisition of more sensitive abdominal imaging modalities by hospitals and clinics has resulted in increased access to this important diagnostic service.^7^ Expectations are that referral rates will continue to increase as obesity and diabetes rates increase, hand‐held ultrasounds become more commonplace at point‐of‐care centers, and NAFLD treatments transition from clinical trials to standards of care.^8^, ^9^

There are a number of possible explanations for the decline in the prevalence of HCV referrals over the past 15–20 years beyond the obvious increase in NAFLD referrals. Perhaps most significant is the introduction of HCV screening of potential blood donors by the Canadian Blood Services in 1992, which resulted in a significant decline in transfusion‐related HCV infections.^10^ Also likely to have contributed are the progressive improvements in antiviral treatments, which serve to decrease the pool of HCV carriers and, thereby, the potential source of infection to others.^11^ Although its efficacy has yet to be documented, in 2008, the local regional Health Authority instituted mobile needle exchange programs with the intent of further decreasing rates of HCV transmission. Whether referral rates for HCV will continue to decline in the near future remains unclear as access to highly potent, well‐tolerated but expensive oral treatment is limited to specialists with expertise in viral hepatitis, and such a policy is likely to contribute to additional referrals, at least in the near future.^12^

Somewhat surprising were the moderate but significant recent declines in the prevalence rates of PBC (APC = −36.4%, 2008/2009–2016/2017) and AIH (APC = −20.8%, 2006/2007–2016/2017) referrals. As with HCV, this decline likely reflects the disproportionate increase in NAFLD referrals. However, the decline in absolute numbers of patients with these conditions parallels that reported for other autoimmune disorders in Canada, which remains unexplained.^13^ Perhaps also contributing to the decline is the appreciation that NAFLD can be associated with positive autoantibody testing.^14^ Thus, some early referrals that had previously been considered PBC or AIH on the basis of the presence of autoantibodies are more recently being correctly diagnosed as NAFLD.

The initial increase and subsequent stabilization of ALD prevalence rates despite the large number of NAFLD referrals remains unexplained. There have been no changes to the availability of alcohol and the socioeconomic status of patients, which can be associated with the extent of alcohol consumption, had not changed during the study period.^15^

Presumably, the significant increase in the mean ages of referred patients over the past 25 years reflects the transition from a predominantly young, viral hepatitis patient population to one of NAFLD subjects whose mean age at diagnosis tends to be in the fifth or sixth decade of life.^16^, ^17^ Also to be considered is the possibility that, over time and with increased knowledge and experience in managing liver disease patients, referring physicians feel more comfortable continuing to manage these patients until they have reached a later stage in their disease course. Unfortunately, the database used for this study does not contain the necessary data (e.g. fibroscan results) required to address that possibility.

There are certain additional limitations to this study that warrant emphasis. First, histological confirmation of diagnoses was limited. Second, the results reflect those of a single center and need to be confirmed at other sites. Third, data are limited to outpatient populations. Finally, the specific liver clinic for this study captures all liver disease patients in the referral area, and therefore, the findings are not specific for an urban or rural practice.

In conclusion, the liver disorders that constitute an outpatient hepatology practice are varied and not predominantly ALD. In addition, there have been significant changes to the prevalence rates of specific liver diseases referred for assessment over the past 25 years. These findings highlight the dynamic nature of hepatology and the need for training and knowledge in various disciplines, including biochemistry, virology, immunology, and addictions medicine amongst others.
